# Insights into the Function and Evolution of Taste 1 Receptor Gene Family in the Carnivore Fish Gilthead Seabream (*Sparus aurata*)

**DOI:** 10.3390/ijms21207732

**Published:** 2020-10-19

**Authors:** Anna Rita Angotzi, Sara Puchol, Jose M. Cerdá-Reverter, Sofia Morais

**Affiliations:** 1 Department of Fish Physiology and Biotechnology, Instituto de Acuicultura de Torre de la Sal, IATS-CSIC, Torre la Sal s/n, Ribera de Cabanes, 12595 Castellon, Spain; sarapuchol@iats.csic.es (S.P.); jm.cerda.reverter@csic.es (J.M.C.-R.); 2 Lucta S.A., Innovation Division, UAB Research Park, 08193 Bellaterra, Spain; sofia.morais@lucta.com

**Keywords:** *T1R* receptors, *G*αi protein, alpha subunit, taste, umami, amino acids

## Abstract

A plethora of molecular and functional studies in tetrapods has led to the discovery of multiple taste 1 receptor (*T1R*) genes encoding G-protein coupled receptors (*GPCRs*) responsible for sweet (*T1R2* + *T1R3*) and umami (*T1R1* + *T1R3*) taste. In fish, the *T1R* gene family repertoires greatly expanded because of several *T1R2* gene duplications, and recent studies have shown *T1R2* functional divergence from canonical mammalian sweet taste perceptions, putatively as an adaptive mechanism to develop distinct feeding strategies in highly diverse aquatic habitats. We addressed this question in the carnivore fish gilthead seabream (*Sparus aurata*), a model species of aquaculture interest, and found that the *saT1R* gene repertoire consists of eight members including *saT1R1*, *saT1R3* and six *saT1R2a*-*f* gene duplicates, adding further evidence to the evolutionary complexity of fish*T1Rs* families. To analyze *saT1R* taste functions, we first developed a stable gene reporter system based on Ca^2+^-dependent calcineurin/*NFAT* signaling to examine specifically in vitro the responses of a subset of *saT1R* heterodimers to L-amino acids (L-AAs) and sweet ligands. We show that although differentially tuned in sensitivity and magnitude of responses, *saT1R1*/*R3*, *saT1R2a*/*R3* and *saT1R2b*/*R3* may equally serve to transduce amino acid taste sensations. Furthermore, we present preliminary information on the potential involvement of the G*i* protein alpha subunits *saGαi1* and *saGαi2* in taste signal transduction.

## 1. Introduction

Perception of sweet, umami and bitter taste is mediated by two distinct *G* protein-coupled receptor (*GPCR*) families, namely by taste 1 receptor (*T1R*) and *T2R*, mostly expressed in taste buds [1,2,3]. In most vertebrates, the *T1R* gene repertoire is relatively conserved and consists of three ancient duplicated genes that diverged before fish/tetrapod diversification, about 400 million years ago (MYA) [4,5]. The *T1R* family’s receptor subunits (*T1R1*, *T1R2* and *T1R3*) dimerize to form functional receptors. The combination of *T1R1*/*T1R3* forms the umami receptor and is activated by amino and nucleic acids, and the *T1R2*/*T1R3* heterodimer responds to natural and artificial sugars including sweet proteins and D-amino acids [6]. Mammalian *T1R* transduction pathways of both sweet and umami sensations are subsequently initiated through multiple *G*-proteins [7,8]. Receptor activation leads to the release of *Gα* gustducin (*Gα*gust) from the beta-gamma subunits (*Gβƴ*) of the *G*-protein complex, and two parallel signaling cascades are then initiated which converge on common steps that mediate a rise in intracellular Ca^2+^ followed by neurotransmitter release. On one hand, *Gβƴ* dimer activates phospholipase *Cβ2* (*PLCβ2*) to generate diacylglycerol and inositol triphosphate (*IP_3_*), which binds to its receptor *IP_3_R* in the endoplasmatic reticulum, triggering the release of Ca^2+^ stores and, finally, *TRPM5* ion channel-mediated membrane depolarization [9,10]. On the other hand, *Gα*gust down-regulates adenylyl cyclases (*ACs*) leading to the inhibition of *cAMP* production. Decrease in *cAMP* levels promotes the downregulation of the *cAMP*-dependent protein kinase A (*PKA*), with consequent activation of *IP3Rs* and *PLCβ2* signaling components, both inhibited by *PKA* in resting states. The upregulation of *IP3Rs* and *PLCβ2* leads to intracellular Ca^2+^ release and downstream membrane depolarization [11,12,13]. Two seminal studies analyzing in silico genomes of fish models indicate that *Gα*gust is not present in teleost fish and other *Gαi* subunits have been proposed to play homologous functions [14,15].

While the existence of unique *T1R1* and *T1R3* genes seems to be a constant feature of vertebrate genomes, numerous *T1R2* paralogs have been thus far identified in several fish species [4,16,17,18,19]. Studies on fish taste function using heterologous expression systems revealed that putative sweet *T1R2* genes respond to L-amino acids (L-AAs) rather than sugars in omnivore species such as zebrafish (*Danio rerio*) and medaka (*Oryzias latipes*) [20], or preferentially to plant-specific fructose in the herbivorous grass carp (*Ctenopharyngodon idellus*) [19]. These observations indicate that *T1R2* gene expansion may have served a key role in the evolution of species-specific taste adaptation to diverse habitats and diets. To further explore this hypothesis, the main purpose of this study was to describe and functionally characterize the first *T1R* gene repertoire in a carnivorous fish, in addition to a species with key relevance in Mediterranean aquaculture, the gilthead seabream *Sparus aurata (sa*). Furthermore, two novel *Gαi* subunits were tested as putative taste-associated proteins alongside the functional characterization of *saT1Rs*, by means of their pharmacological responses to different L-AAs and sweet tastants.

This information, besides its potential significant value for further clarifying evolutive aspects, is of great practical interest for aquaculture production. Linked to its important role in identifying nutrients and sources of metabolic energy, the *T1R* gene family is associated with the perception of attractive taste modalities. Therefore, understanding the molecular mechanisms that control feeding preferences and promote feed consumption by attractive taste sensations will contribute towards the production of more efficient species-tailored feeds, enabling a better utilization of the diets through the modulation of feeding behavior and food intake. Such knowledge may be particularly relevant during periods of depressed appetite associated to specific physiological or productive stressful events, or when using alternative protein ingredients with low palatability in fish feed formulations.

## 2. Results

### 2.1. Identification of saT1R Genes

Five *saT1R* genes were initially identified through blast searches of the preliminary seabream genome assembly (http://biocluster.her.hcmr.gr/myGenomeBrowser?portalname=Saurata_vI), subsequently cloned in their complete coding sequences [*saT1R1* (846 AAs, XP_030277517), *saT1R2a* (826 AAs, XP_030278006), *saT1R2b* (824 AAs, XP_030278002), *saT1R2c* (817 AAs, MT892735), *saT1R3* (856 AAs, XP_030274769)], and used in the pharmacological characterization of different receptor combinations (all except *saT1R2c*). During the final stages of this study, the genome assembly project was released on NCBI genebank [21] and a new blast search identified a total of seven *saT1R*-related complete coding sequences (CDS) derived from automated computational analysis (Gnomon). The seven sa*T1R* genes included four out of the five initially cloned genes in the present study and three additional *saT1R* gene predictions, hereby referred to as *saT1R2d* (826 AAs, XM_030422143), *saT1R2e* (824 AAs, XM_030422144) and *saT1R2f* (821 AAs, XM_030422145)*,* respectively. Therefore, information available up to date indicates that the *saT1R* gene repertoire comprises eight members: two one-to-one orthologs of *T1R1* and *T1R3* vertebrate genes (*saT1R1* and *saT1R3*) and six *saT1R2* duplicate members (*saT1R2a-f*) highly conserved to each other (nt identity from 83 to 92%), and moderately with respect to *saT1R1* and *saT1R3* (± 47% and 42% respectively). InterProScan scanned against InterPro’s signatures [22] *saT1R* protein alignments show the characteristic conserved domain architecture of family 3 (or *C*-type) *GPCRs* (Figure S1). This includes a large extracellular region containing the N-terminal, the Venus Flytrap (VF) and the 9-cystein-rich (CR) domains that structurally link to the heptahelical transmembrane (7TM) domain located upstream of the intracellular carboxyl-terminal domain [23,24].

### 2.2. Phylogenetic Analysis of saT1Rs and saGαi Genes 

**Figure 1:**
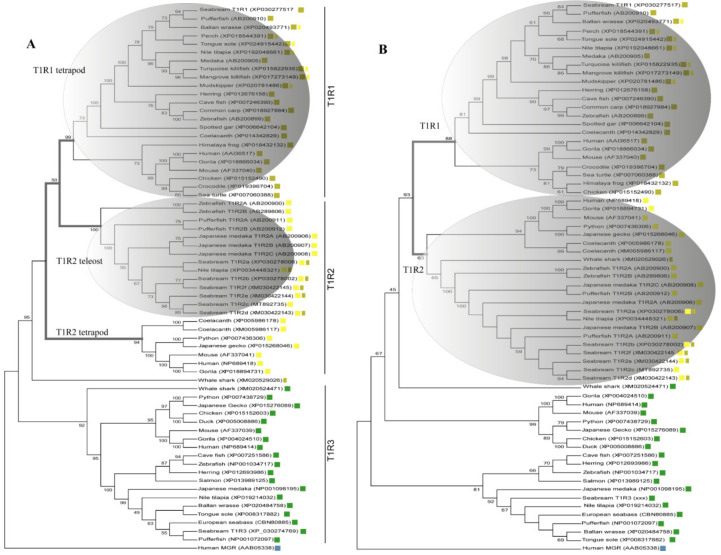
Phylogenetic relationships inferred by Maximum Likelihood (ML) method of CDS-(**A**) and VFD-(**B**) trees using multiple alignments of deduced AA sequences of vertebrate *T1Rs* ortholog genes. Both evolutionary reconstructions cluster *saT1R1*, *saT1R2a_/_b_/_c_/_d_/_e_/_f* and *saT1R3* genes in three distinct clades (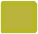 *T1R1*; 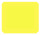 *T1R2*; 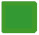 *T1R3*) each comprising their respective ortholog genes from mammals, amphibians, birds and reptiles. The (**A**) topology sets teleost *T1R2* genes evolutionarily closer to *T1R1* than *T1R2* of tetrapod, while two compact *T1R1* and *T1R2* vertebrate clades are observed in the VFD (**B**) topology (grey line branches 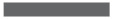). 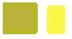 *T1R1* fish AA sequences as deduced from our phylogenetic reconstructions and NCBI-automate-annotated as vertebrate *T1R2* orthologs. 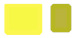 *T1R2* fish AA sequences as deduced from our phylogenetic reconstructions and NCBI-automate-annotated as vertebrate *T1R1* orthologs. Robustness of the trees was estimated by 1000 random bootstrap replications. Only bootstrap values higher than 50% are shown. The human Metabotropic Glutamate Receptor 1 (*MGR1*; 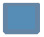 ) was used for rooting the trees. GenBank *T1Rs* accession numbers are indicated next to species names.

Inference of *saT1R* evolutionary relationships was performed using protein datasets including either the complete coding sequence (CDS-Tree) or the Venus Flytrap Domain (VFD-Tree) of annotated *T1R* ortholog sequences (64 sequences from 29 organisms). *T1R* phylogenetic trees from both CDS-Tree and VFD-Tree datasets, support the monophyly of *T1R* paralogs, with *saT1R1*, *saT1R3* and *saT1R2* genes clustering into separate clades, each comprising orthologs from birds, amphibians, reptiles and mammals (Figure 1A,B; Datasets S1, S2). 

Although the phylogenetic inferences deduced from CDS- and VFD-trees are generally in good agreement, the CDS-Tree sets tetrapod *T1R1* and teleost *T1R2* genes as sister groups (Figure 1A), while a compact *T1R2* vertebrate clade is observed in the VFD-tree topology (Figure 1B).

**Figure 2:**
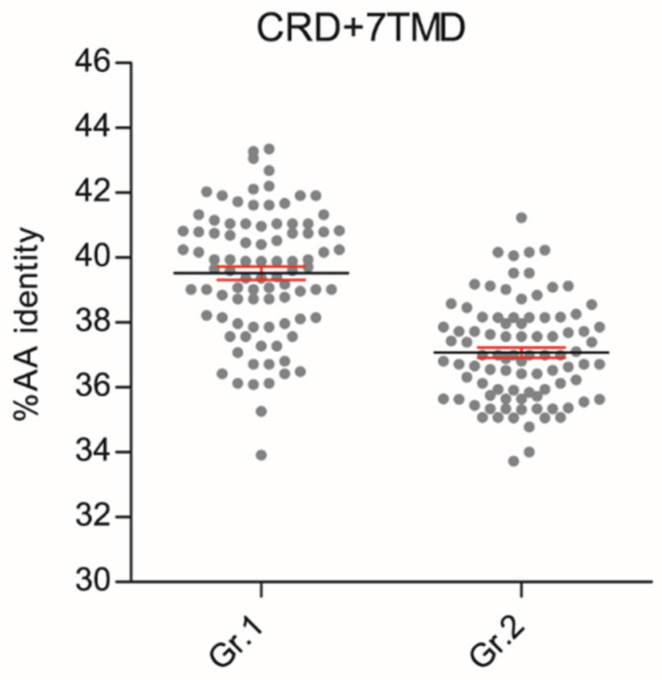
Scatter plots of amino acid conservation within the 9-Cystein-Rich and heptahelical transmembrane domains (CRD + 7TMD, ± 348 AAs) of fish *T1R2* versus tetrapod *T1R1* (Gr.1) or tetrapod *T1R2* (Gr.2). Each dot represents the percentage of AA sequence identities calculated in one-to-one combinatorial arrangements (*n* = 90) of fish *T1R2s* (*n* = 13) with *T1R1* (*n* = 7) or *T1R2* (*n* = 7) tetrapod sequences. T-test analysis of variance followed by Mann Whitney test was implemented to show significant differences between the two groups (*p* < 0.0001); percentage AA identity mean (black line) ± SEM (in red).

These phylogenetic reconstructions suggest that sequence conservation driving phylogenetic clustering of fish *T1R2* with *T1R1* tetrapod may be located outside the VFD. To substantiate this hypothesis, we carried out comparative sequence conservation analyses within the cysteine-rich and heptahelical transmembrane domains (CRD + 7TMD) of several *T1R1* and *T1R2* genes (Figure 2; Dataset S3). This analysis shows that fish *T1R2* have significantly higher AA identity to *T1R1* (39.52% ± 0.91) than to *T1R2* (37.08% ± 0.58) of tetrapods (*p* < 0.0001) in these regions.

Moreover, our phylogenetic analyses refine the accuracy of fish *T1R* annotations, establishing new phylogenetic relationships for previously automatically annotated *T1R1* and *T1R2* fish sequences that unambiguously fall into opposite *T1R* orthology clades in both CDS- and VFD- datasets. 

**Figure 3:**
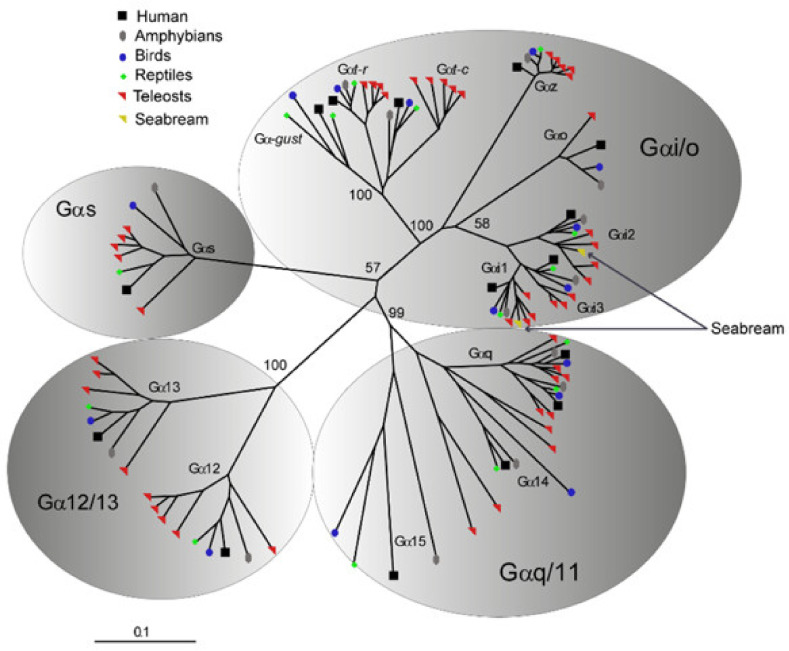
Radial view representation of an unrooted Neighbor-Joining tree depicting the phylogenetic relationship among (*Gα*) protein subunits of *Gs*, *Gi*/*Go*, *Gq*/*G11* and *G12*/*G13* classes of representative vertebrate homologs. *saGαi1* and *saGαi2* AA sequences cluster in one-to-one ortholog relationships within the corresponding *Gαi* monophyletic clade, suggesting *saGαi1-2* duplicated origin from ancient vertebrate whole genome duplications. Numbers above nodes indicate bootstrap values (based on 1000 replicates) that support the respective branch. The scale (lower left corner) indicates the mean number of AA substitutions per site.

Phylogenetic analyses were additionally performed to validate the identity of *saGαi1* and *saGαi2* genes retrieved from NCBI (acc.ns. XP_030281956 and XP_030276216, respectively) and used for the in vitro transfections. Orthology inferences of *saGαi* CDSs were assessed using several vertebrate *Gα*-protein subunits (111 sequences from 18 organisms), representative of the four major classes *Gs*, *Gi*/*Go*, *Gq*/*G11* and *G12*/*G13* [25]. Results confirmed that *saGαi1* and *saGαi2* genes are members of the *Gαi*/*o* class, clustering closely with the respective vertebrate orthologs (Figure 3; Dataset S4).

### 2.3. Validation of pGL3-NFAT-luc *Reporter* Constructs for Intracellular Ca^2+^ Quantification in Transiently Transfected HEK293 Cells

**Figure 4:**
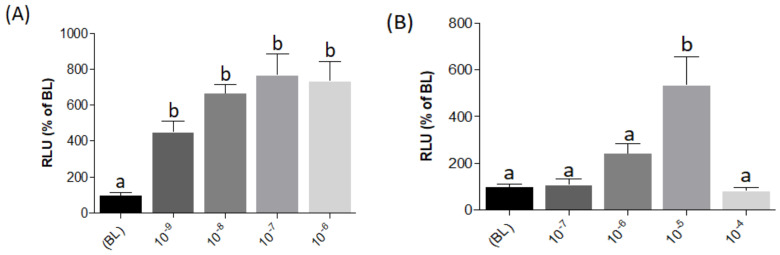
Evaluation of pGL3-*NFAT*-luc construct responsiveness to 10-fold serial dilutions to PMA (**A**) and ionomycin (**B**) in transiently transfected HEK293 cells. RLU%: Relative Luminescence Unit percentage. Evaluation of L-AA responses was based on the RLU mean ± SEM of four independent determinations normalized to the mean response of the same transfections (*n* = 4) stimulated with assay medium (Dulbecco’s Modified Eagle Medium (DMEM) + 1% fetal bovine serum (FBS)). BL (Basal Levels) = 100% RLU. Different lowercase letters (a,b) on top of bars indicate significant differences (*p* < 0.05) between concentrations, assessed by one-way ANOVA followed by Tukey’s Multiple Comparison Test (GraphPad Prism version 5.00 Software, La Jolla, CA, USA).

The luciferase reporter system used in the present study is based on a pGL3-plasmid including three (N)uclear (F)actor of (A)ctivated T cells (*NFAT*)-responsive elements located upstream of an interleukin-2 (*IL2*) minimal promoter driving expression of the firefly luciferase reporter gene (pGL3-*NFAT*-luc plasmid; Addgene (Watertown, MA, USA), cat. number. 17870). To evaluate pGL3-*NFAT*-luc construct responsiveness, transiently transfected HEK293 cells were stimulated with Phorbol 12-myristate 13-acetate (PMA) and ionomycin, two compounds known to modulate *NFAT* signaling by mobilization of intracellular Ca^2+^ storages [26,27,28,29]. Both compounds showed significant luminescence dose-response increases, indicating pGL3-*NFAT*-luc construct effectiveness for intracellular Ca^2+^ quantification (Figure 4A,B; Datasets S5 and S6) when expressed in a HEK293 heterologous cell system. 

### 2.4. Validation of Stable pGL3-NFAT-Luc-HEK293 Cell Lines

**Figure 5:**
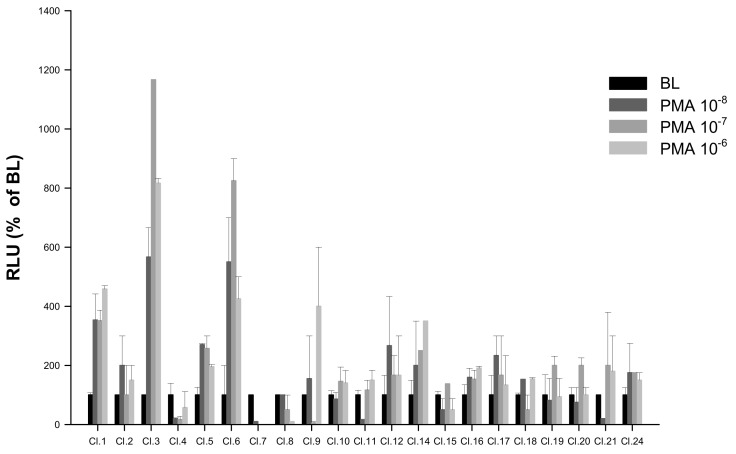
Screening of putative stable pGL3-*NFAT*-luc-HEK293 clones by response to PMA (*n* = 4) at 10-fold serial dilutions tested over three concentrations (10^−8^, 10^−7^ and 10^−6^). Evaluation of PMA responses was based on the RLU mean ± SEM of four independent determinations normalized to the mean response of the same transfections stimulated with assay medium (DMEM +1% FBS). BL (Basal Levels) = 100% RLU. Clone 3 (Cl.3) was the most responsive to PMA-luminescence induction.

HEK293 cells lines stably expressing pGL3-*NFAT*-luc reporter construct were generated as described in Section 4.3. Among the 22 potentially stable pGL3-*NFAT*-luc-HEK293 clones screened by PMA, the clone showing highest luminescence responses (Cl.3) was selected for subsequent *saT1Rs* transient transfections (Figure 5; Dataset S5). 

### 2.5. In Vitro Characterization of saT1: Responses to L-AAs 

Consistent with the view that *T1R* proteins function as heterodimeric complexes, co-expression of *T1R3* with either *T1R1* or *T1R2* has been largely reported in taste receptor cells of both mammals and fish [17,30,31]. Additionally, in vitro functional studies have been performed in several species, where it is generally assumed that the extent of T1R activation following L-AA and sugar stimuli is positively correlated to taste sensations [20,32,33]. However, as no information is available thus far on the activity of T1R receptors in a carnivorous fish species, we addressed this knowledge gap by characterizing the pharmacological responses of heterodimeric *saT1R1*/*R3*, *saT1R2a*/*R3* and *saT1R2b*/*R3* following transient transfections in stable (Cl.3) cell lines. Before cell culture experiments, expression of the initially identified *saT1R* genes was verified by qPCR and RT-PCR screening in several putative taste and non-taste tissues, and given that no evidence of *saT1R2c* transcriptional activity could be found (Figure S2), this *saT1R2* duplicate was not considered for further functional investigation.

**Figure 6:**
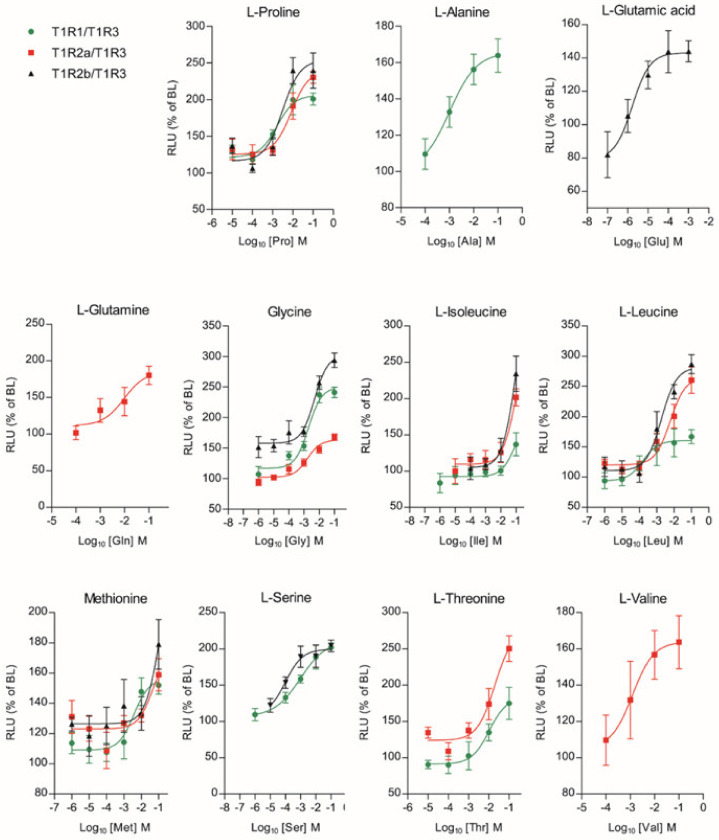
Amino acid Log-dose response curves of activation in heterologous co-transfected (Cl.3) cells with *saT1R1/3* (green dots), *saT1R2a/3* (red squares) and *saT1R2b/3* (black triangles) heterodimers. Evaluation of L-AA responses was based on the RLU mean ± SEM of four independent determinations normalized to the mean response of the same transfections stimulated with reduced assay medium (Basal Medium Eagle w/o L-glutamate (BME) + 1% FBS), and expressed as the percentage relative to basal levels (BL).

Among the full set of 20 L-AAs that were tested up to eight concentrations (10 nM to 100 mM)*,* 11 of them generated a dose-response activation (Glutamic acid (Glu); Glutamine (Gln); Proline (Pro); Glycine (Gly); Alanine (Ala); Threonine (Thr); Serine (Ser); Valine (Val); Isoleucine (Ile); Leucine (Leu); Methionine (Met)), for at least one of the three *saT1R* heterodimers (Figure 6; Dataset S5).

**Figure 7:**
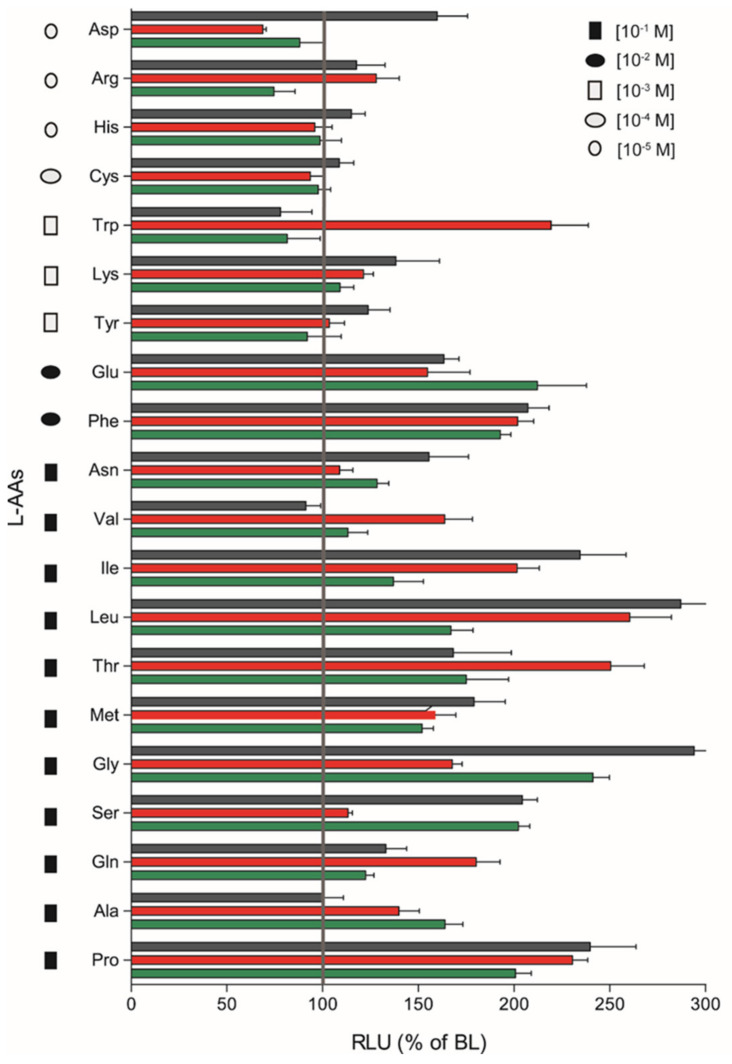
Magnitudes of *saT1R1*/*R3*, *saT1R2a*/*R3* and *saT1R2b*/*R3* responses (green, red and black bars, respectively) to L-AAs based on RLU recorded at maximal stimulation dosages (MSD) within molar range concentrations of (10^−1^–10^−5^).

The remaining nine L-AAs (Arginine (Arg); Aspartic acid (Asp); Histidine (His); Asparagine (Asn); Cysteine (Cys); Lysine (Lys); Tryptophan (Trp); Tyrosine (Tyr); Phenylalanine (Phe)) did not respond in a dose-response manner, although significant stimulatory effects were observed for some heterodimers at a given AA concentration by plotting the magnitude of responses of *saT1R1*/*R3*, *saT1R2a*/*R3* and *saT1R2b*/*R3* at the maximal stimulation dosage (MSD) for each of the 20 L-AAs (Figure 7).

We evidenced fairly promiscuous profiles of activation, which suggests that the three taste-subunit combinations may equally serve to transduce L-AA taste sensations. *saT1R2b*/*R3* was generally the most responsive heterodimer, showing the highest responses to Asp, His, Cys, Lys, Tyr, Phe, Asn, Ile, Leu, Met, Gly, Ser and Pro stimulations, followed by *saT1R2a*/*R3* (Arg, Trp, Val, Thr, Gln) and *saT1R1/R3* (Glu, Ala) (Figure 7; Table S1).

**Figure 8:**
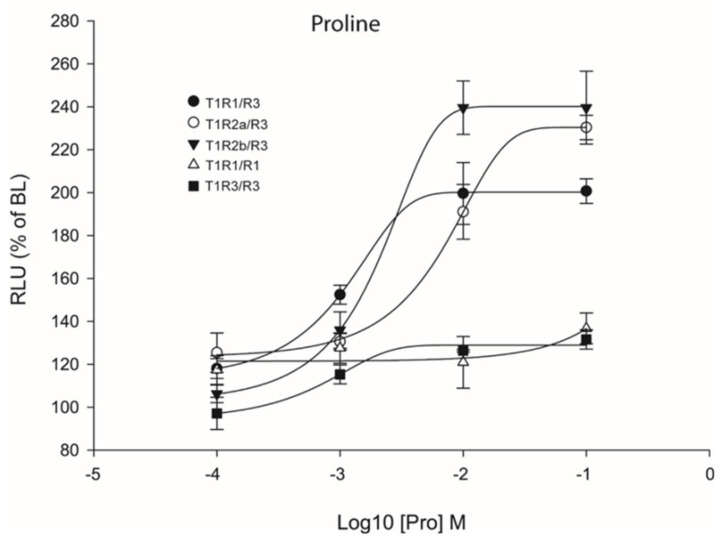
Comparisons of proline Log-dose response curves of activation in heterologous transfections of hetero- and homo-dimeric *saT1R* combinations of *saT1R1*/*R3* (black dots), *saT1R2a*/*R3* (white dots), *saT1R2b*/*3* (black triangles), *saT1R1*/*R1* (white triangles) and *saT1R3*/*R3* (black squares). Response is measured as RLU mean ± SEM of four independent determinations normalized to the mean response of the same transfections stimulated with reduced assay medium (BME + 1% FBS), expressed as percentage relative to basal levels (BL).

Previous studies have reported that heterodimeric coupling of *saT1R* complexes may not be the only mode of functional activation since these subunits, especially *T1R3*, can also couple as homodimers [30,34,35,36]. To analyze this possibility, transient transfections with double amounts of *saT1R3* alone (or of *saT1R1*, as additional control) were also performed using L-Pro stimuli, as it presented highly reproducible dose responses for all three *saT1R* heterodimers (Figure 8; Dataset S5). 

Our results showed that Cl.3 cells transiently transfected with only one *saT1R* subunit type did not respond to Pro stimulation. In addition, to reject the possibility that L-AA ligands could induce taste receptor-independent rises of Ca^2+^ [37], negative controls were also implemented by transfecting empty pcDNA™3 constructs, and potential non-specific luminescence signals were estimated at known MSDs for a subset of L-AAs (Pro, Ala, Gln, Ser, and Val) at 100 mM (Figure S3).

Furthermore, evaluation of half-maximal effective concentrations (EC50) of the L-AAs showing dose-response curves indicates important differences in the sensitivity of *saT1R1*/*R3*, *saT1R2a*/*R3* and *saT1R2b*/*R3*. Although in the majority of cases direct comparison for a given L-AA was not possible for the three heterodimers, *saT1R2b*/*R3* responded with the highest EC50 sensitivity recorded (Glu 1.68 × 10^−6^ and Ser 9.94 × 10^−5^ M, respectively*;* Table 1). 

On the other extreme, *saT1R2a*/*R3* generally responded with the lowest sensitivity to L-AAs. Overall, and irrespectively of the specific heterodimer that was assessed, the following molar rank order of L-AA potencies as activators of *saT1R*-mediated taste responses was found*:* Glu > Ser > Leu > Ala > Pro > Val > Gly > Met > Gln > Thr > Ile (Table 1). It is noteworthy that the majority of EC50 sensitivity values recorded (in the range of 10^−3^-10^−4^ M) are in good agreement with those previously reported for T1R heterodimeric complexes of zebrafish and medaka using Ca^2+^-sensitive fluorescent dyes [20], further validating our methodological choice based on luciferase gene reporter systems.

### 2.6. In Vitro Characterization of saT1R: Responses to Natural Sugars

**Figure 9:**
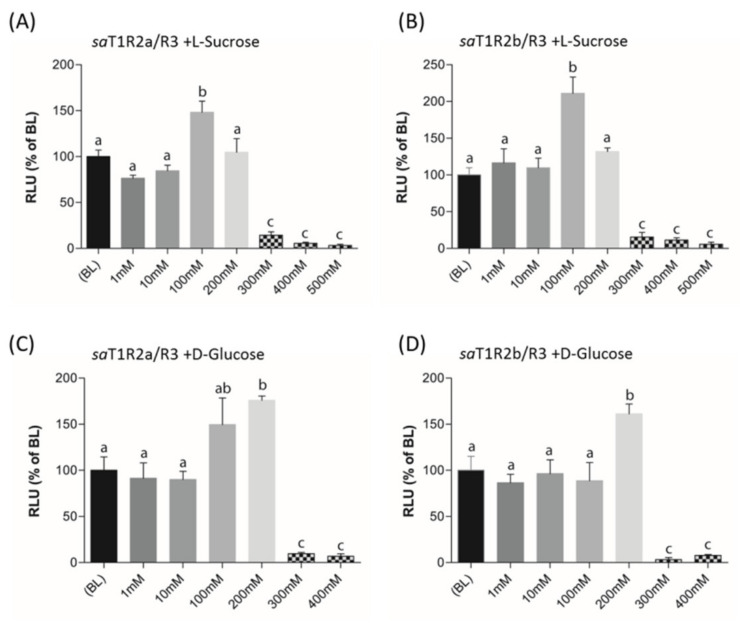
*saT1R2a*/*R3* and *saT1R2b*/*R3* heterodimer responses to L-Sucrose (**A**,**B**) and D-glucose (**C**,**D**) at different concentrations (1–500 mM). Evaluation of sugar responses was based on the RLU mean ± SEM of four independent determinations (*n* = 4) normalized to the mean response of same transfections stimulated with reduced assay medium (BME + 1% FBS). BL (Basal Levels) = 100% RLU. Different lowercase letters (a,b,c) on top of bars indicate significant differences (*p* < 0.05) between concentrations, assessed by one-way ANOVA followed by Tukey’s Multiple Comparison Test (GraphPad Prism version 5.00).

When L-Sucrose and D-Glucose were tested as potential ligands of putative sweet taste receptors *saT1R2a*/*R3* and *saT1R2b*/*R3*, they elicited dose response patterns similar to those observed in mammalian *T1R2*/*R3* [32,38]. Significant activations of both *saT1R2a*/*R3* and *saT1R2b*/*R3* were observed at 100 or 200 mM for L-Sucrose (Figure 8B, Figure 9A; Dataset S7) or D-Glucose (Figure 9C,D; Dataset S7), respectively, while at higher doses (300–500 mM), both sugars were toxic for the cells and/or inhibited their stimulation.

### 2.7. Pharmacological Responses to Proline in the Presence of saGαi1-2

**Figure 10:**
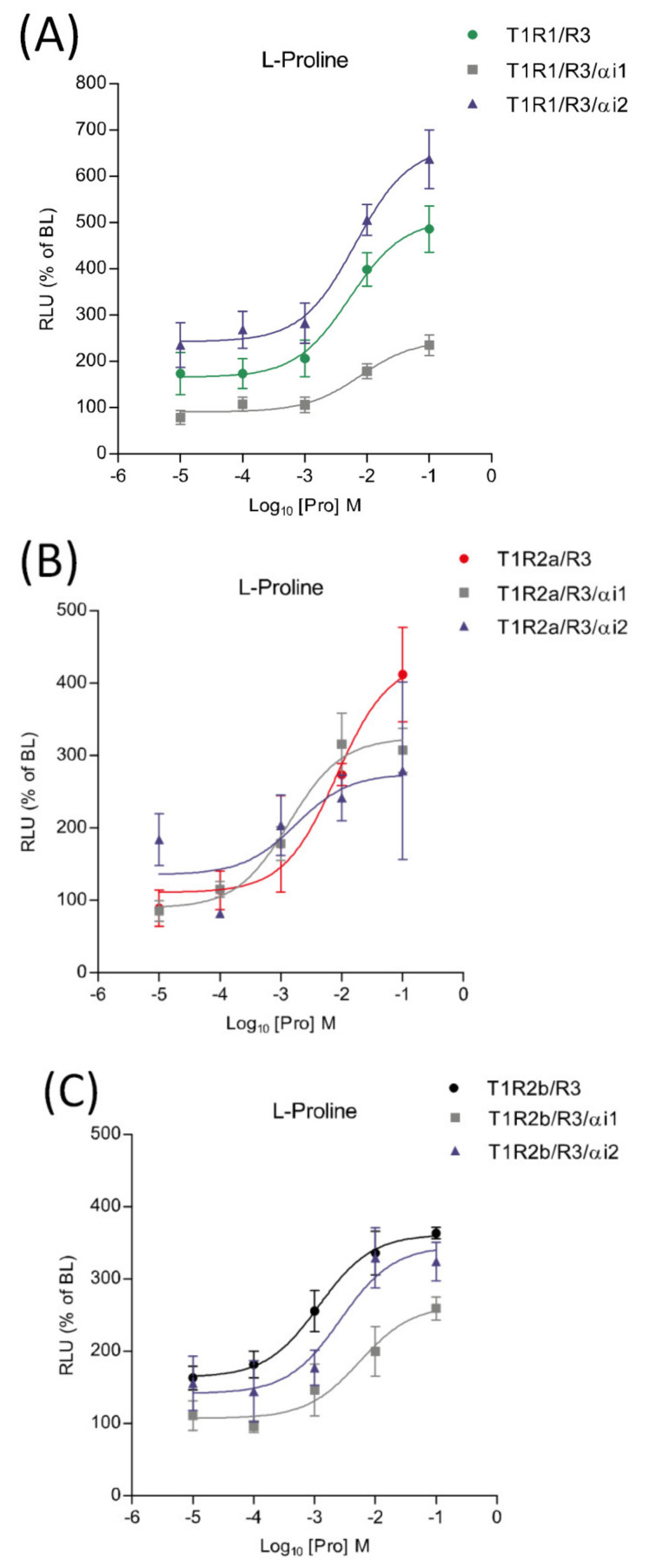
Comparisons of proline Log-dose response curves of activation in heterologous transfections of *saGαi1* and *saGαi2* in combination with *saT1R1*/*R3* (**A**), *saT1R2a*/*R3* (**B**) and *saT1R2b*/*3* (**C**). Evaluation of proline responses was based on the RLU mean ± SEM of four independent determinations normalized to the mean response of same transfections stimulated with reduced assay medium (BME + 1% FBS), expressed as the percentage relative to basal levels (BL).

The potential involvement of *saGαi1-2* subunits in taste signaling pathways was investigated using transient heterologous expression of *saT1R1*/*R3*, *saT1R2a*/*R3* and *saT1R2b*/*R3* heterodimers in combination with *saGαi1* or *saGαi2* constructs, and using Pro as a standard ligand. When co-transfected with the *saT1R1*/*R3* heterodimer, the two subunits triggered opposite effects: stimulatory in the case of *saGαi2* and inhibitory for *saGαi1* (Figure 10A; Dataset S5). On the other hand, both *saGαi1-2* mediated inhibitory effects when co-transfected with the *saT1R2b*/*R3* heterodimer (Figure 10C; Dataset S5). Due to the high variability recorded between independent determinations (*n* = 4), no clear profiles were observed for *saT1R2a*/*R3*/*αi1* or /*αi2* combinations, although both *saGαi* subunits show a general tendency towards inhibitory effects (Figure 10B; Dataset S5). 

## 3. Discussion

The phylogenetic tree reconstructions reported new putative *T1R* sequences identified in the present research. All ortholog proteins for each of the three *T1R* ancient paralogs showed similar conserved domains, as evidenced by their phylogenetic relationships. The earlier evolutionary emergence of T1R3 suggests that it might have been the first duplicated gene from a common T1R3/T1R1-2 ancestor.

The presence of multiple *T1Rs* in both fish and tetrapod genomes indicates that *T1R* duplications might have occurred before Actinopterygian/Sarcopterygian divergence, around 400 MYA [39]. Blast searches in ancient vertebrate genomes of *Ciona intestinalis* amphioxus, hagfish and lampreys failed to detect *T1R* distant homologs, while an elasmobranch genome (whale shark) exhibits two *T1R* genes. This suggests that the evolutionary expansion of *T1R* gene family might have taken place after jawless vertebrate radiation, predating the osteichthyes/chondrichthyes divergence. Furthermore, *saT1R2* paralogs fall into a major clade including multiple *T1R2* orthologs from other teleost species, suggesting that fish *T1R2* expansion might have occurred in the common ancestor of extant teleost, in line with the teleost-specific (TS)–Whole Genome Duplication (WGD) hypothesis [40,41]. Yet, the presence of two *T1R2* paralogs in the primitive land-vertebrate coelacanth, and the general consensus for singleton *T1R1* and *T1R3* loci in teleost genomes, also opens up alternative scenarios for an ancient *T1R2* gene-specific duplication event predating Actinopterygii/Sarcopterygii divergence.

Both *T1Rs* phylogenies as deduced from CDS- and VFD-trees have been previously described [4,18], and possible functional implications can be ascertained based on these tree reconstructions. 

The pivotal role of the class *C*-*GPCR*-VFD in ligand binding and transduction activation has been long recognized since the first solved crystal structure of the human *MGR1*. This protein is characterized by a dimeric bi-lobed protomer organization of the VFD domain that forms either an open “V” or a compact “U” dimer arrangement, reflecting resting or active states, respectively, upon glutamate binding [42]. Although attempts to purify and crystallize mammalian T1R-VFD have been unsuccessful until now, *T1Rs* are also likely to have a dynamic rearrangement of the dimeric VFD, based on sequence and protein structure similarities to *MGR1*. Recently, determination of the crystal structure of the heterodimeric *T1R2a*/*3*-VFDs in medaka indicated similar conformational changes as those underling human *MGR1* activation [43], suggesting that the molecular structural basis for ligand recognition is presumably conserved within class *C*-*GPCRs* [44]. Moreover, protein modeling of medaka *T1R2a*/*3*-VFDs shows that ligand domains in this module might bind to different amino acids in a broad yet discriminating manner [45].

Although orthosteric sites of class *C*-*GPCR* have been thus far mainly identified in the VFD, the CRD and 7TM domains are also known to provide additional molecular basis for receptor activation via allosteric binding to endogenous and synthetic ligands, enabling to change the conformational state of the receptor, and thereby potentially modulating affinities and/or efficacies of orthosteric ligands [46]. For instance, mutational analyses and molecular modeling studies showed that several allosteric modulators bind to conserved pockets located in the CRD and 7TM domains of metabotropic, calcium sensing and sweet taste receptors [47,48,49,50,51].

Our *T1R* phylogenetic reconstructions based on CDS and VFD sequence datasets indicate that sequence conservation driving phylogenetic clustering of fish *T1R2* with *T1R1* tetrapod resides outside the VFD. Indeed, comparative sequence conservation analyses of (CRD + 7TMD) domains located downstream of the VFD show that fish *T1R2* have significantly higher AA identity to *T1R1* than to *T1R2*, suggesting that orthosteric and/or allosteric *T1R2* responsive domains for L-AA recognition in fish might reside in these regions. To validate these predictions, dissection of conserved domains and generation of truncated versions of fish *T1Rs* should be performed to elucidate ligand binding specificity of individual protein modules. Such information would enable a better understanding of the structure-to-function link at the basis of umami and sweet *T1R*-mediated taste modalities in fish.

Evaluation of *saT1Rs* pharmacological responses to L-AAs and sweet ligands was conducted by systematic transient transfections of the heterodimers *saT1R1*/*R3*, *saT1R2a*/*R3* or *saT1R2b*/*R3* in HEK293 cells lines stably expressing pGL3-*NFAT*-luc reporter construct for intracellular Ca^2+^ /*NFAT* signaling detection. The basic principle in the use of *NFAT* luciferase constructs for quantification of *saT1R* responses lies in the role of Ca^2+^ as major secondary messenger in both taste transduction [52,53,54] and *NFAT*-mediated immune responses [55]. *NFAT* proteins reside ubiquitously in the cytosol in their inactive phosphorylated state [56]. Upon cell stimulation, increase of the Ca^2+^-dependent calcineurin dephosphorylates the serine-rich regions of *NFAT* regulatory domains, resulting in nuclear *NFAT* translocation and DNA binding to initiate and maintain different specific transcriptional programs [57,58,59,60]. Over the past decade, taste receptor functions have been mainly addressed by in vitro assay systems using Ca^2+^-sensitive fluorescent dyes, such as fluo-3, fluo-4 and fura-2 [20,38,61,62], or luminescence-based assays using jellyfish-derived apophotoproteins, such as aequorin, obelin and clytin as Ca^2+^ indicators [63], and more recently, the evaluation of taste receptor induced-calcium signaling in cell culture systems has been strengthened by the implementation of Förster resonance energy transfer (FRET) technologies [43,45]. To our knowledge, this is the first report employing a luciferese gene reporter system based on Ca2^+^-dependent calcineurin/*NFAT* signaling for taste receptor functional assays.

The in vitro characterization of *saT1R* responses to L-AAs and natural sugars indicate that *T1R2*-mediated sweet taste signaling has been conserved through the divergence between tetrapod and fish lineages. Nevertheless, *T1R2* genes of some fish species have additionally acquired the ability for sensing AA compounds with considerably higher sensitivity than sugars, possibly as an adaptive mechanism to diversify feeding habits. In support of fish *T1R2* adaptive functions, it was recently shown that a subset of four recently duplicated *T1R2* paralogs in the herbivorous grass carp displayed enhanced *T1R2s*/*T1R3* responses to plant-specific fructose. The authors suggest that *T1R2* gene expansion in this species (possibly deriving from the extra lineage-specific genome duplication of cyprinids [64]) underlies taste adaptive strategies to dietary transition from carnivore to herbivore food habits [19]. Similarly, heterologous transfections of *T1R2a*/*b*/*c*/*R3* dimeric complexes and testing with a broad range of L-AAs and natural and artificial sugars as potential ligands in the omnivorous zebrafish and medaka fish suggested that duplicated *T1R2s* in these species may have evolved for tuning a wide range of sensory modalities with a prominent sensitivity to amino acids [20]. Our data in a carnivorous species also support this idea. 

Hence, current evidence suggests that expansion of *T1R2* paralogs in fish genomes may have been particularly prone to positive selection, acting to improve fitness advantage in feeding adaptations to natural environments. This is particularly relevant in fishes, which are the largest and most diverse group of vertebrates, with nearly 30,000 species accounting for approximately half of all extant vertebrates [65,66]. Furthermore, fish inhabit almost every aquatic environment, many sharing the same ecological niche, often leading to the evolution of feeding specializations, for which variability in dietary preferences is key [67]. Other important fish gene duplications conferring adaptation to a wide variety of habitats have been reported for several gene families including, for instance, opsins [68,69], detoxification sulfotransferase (*SULT*) genes [70] or antifreeze glycoproteins (*AFGP*) [71], among others.

The development of a taste system with a broad spectrum and high sensitivity to detect amino acids in fish species is logically linked to the particularly high reliance on proteins rather than carbohydrates as a main source of metabolic energy, while glucose to satisfy the animal’s needs is produced mostly through gluconeogenesis from amino acids [72]. Such high protein requirements of fish are manifested by a striking 50–300% higher optimal dietary protein levels in aquaculture fish diets compared to terrestrial farm animals [73]. Within this high range, protein and AA requirements of different fish species can vary greatly; herbivorous and omnivorous species may require a diet with 25 to 35 percent crude protein, while carnivorous fish generally need higher amounts ranging from 35 to 50 percent of the total diet [74,75,76]. Furthermore, fish species can be very efficient in utilizing dietary amino acids for endogenous protein synthesis and deposition into body with high rates [77]. Nevertheless, precisely formulating diets in accordance to the species’ specific amino acid dietary requirements is a critical aspect given that AA deficiencies, or excesses, can impair key metabolic pathways, body homeostasis, immune responses, reproduction, welfare and growth [78]. In addition, importantly, high quality protein sources and supplemental AAs are expensive feed components that greatly influence production costs of global intensive aquaculture systems. An efficient utilization of dietary protein not only depends on its amino acid profile meeting a specific species’ life stage or physiological state requirements, but it is equally important to ensure a good acceptability of the feed and design appropriate feeding regimes that minimize feed waste. The need to substantially replace fish meal by alternative (non-capture fisheries based) protein sources in fish diets is a major focus of modern-day aquaculture [79,80]. Experimenting with mixtures of a wide range of alternative protein ingredients, combined with crystalline amino acid supplementation, has enabled great advances in this sustainability target. However, this is not always possible to accomplish in a cost-effective manner, particularly in carnivorous fish species. Difficulties are often associated to a lower acceptability (i.e., reduction of feed intake) of alternative proteins, particularly those of vegetable origin [81,82,83]. In this respect, an improved understanding of fish taste palatability and preferences will contribute towards achieving optimized feeding formulations tailored to species of aquaculture interest. 

Two new *saGαi* genes have been additionally reported and phylogenetically characterized in this study. Taken together, our results suggest that despite their primary sequence conservation (83% identity), duplication of these *saGαi* genes might have provided genetic background for functional diversification. Furthermore, we provide preliminary data supporting the differential involvement of *saGαi1* and *saGαi2* subunits in taste transduction signal. While enhancement of the *saGαi*-mediated stimulatory effects on *T1Rs* are in good agreement with the known action of mammalian *Gα*gust in taste chemosensory transduction [34,84], it is challenging to speculate on the possible mechanisms involved in *saGαi*-mediated inhibitory effects. This certainly needs to be explored through further research, but existing literature enable us to formulate a hypothesis that could potentially explain these preliminary results. *GPCRs* have a functional versatility that enables them to activate more than one *G* protein type to change dynamically downstream signaling networks. For instance, the stimulatory α-subunit (*Gαs*) of the ubiquitously expressed *Gs* protein is known to have opposite effects to *Gαi* by mediating the activation of *ACs*, resulting in increases of intracellular *cAMP* and activation of *PKA* with consequent downregulation of *PLCβ2* and *IP3R* components leading to declining Ca^2+^ levels [85,86]. In the context of our experiments, we can speculate that in vitro *saGαi1* or *saGαi2* overexpression might cause, directly or indirectly (and depending on the *saT1R* heterodimer type), functionally-impaired modulation of alternative *Gα* subunit proteins (such as *Gαs*), leading to intracellular Ca^2+^ decreases (as deduced from the reduced RLU levels). Indeed, recent studies based on computer modeling and bioluminescence FRET assays provided evidences for different *Gαi* and *Gαs* interacting interfaces in *GPCR* heteromeric complexes [87]. Furthermore, functional studies demonstrated the existence of cross-regulation leading to opposite downstream signals between *Gi* and *Gs* pathways [88].

## 4. Materials and Methods 

### 4.1. In Silico Identification and Molecular Cloning of saT1Rs and saGαi Genes

In the initial phase of the study, pufferfish (*Tetraodon negroviridis*) *T1R1* protein sequence (acc.no. AB200910) was used as a query against the preliminary seabream genome assembly (http://biocluster.her.hcmr.gr/myGenomeBrowser?portalname=Saurata_vI [21]), by translated tblastn search algorithm. Five unique *saT1R* gene fragments were found scattered throughout different contigs (Scaffold 199W17253) and subsequently analyzed using bioinformatics tools to predict coding sequences (https://blast.ncbi.nlm.nih.gov/Blast.cgi; http://web.expasy.org/translate), determine intron-exon boundaries (http://genes.mit.edu/GENSCAN.html), build alignments to identify multiple paralogs (https://www.ebi.ac.uk/Tools/msa/clustalo) and predict signal peptides as primary indication of functional genes (http://www.cbs.dtu.dk/services/SignalP). In silico *saT1R* gene fragments were identified as orthologs of medaka and zebrafish *T1R1*, *T1R2a*, *T1R2b*, *T1R2c* and *T1R3* genes [20]. Four out of the five fragments (all except *T1R2c*) were further extended by 5′ and 3′ rapid amplification of cDNA ends (RACE) libraries prepared from tissue pools of lip, tongue, oral cavity epithelium and gill mRNAs (SMARTer ^®®^ RACE 5′/3′ Amplification Kit, Clontech, Mountain View, CA). Amplification of *saT1R* CDS was performed using Long-Range PCR Kit (Qiagen, Toronto, CA); RT-PCR yields were subsequently gel purified (QIAquick, Qiagen, Toronto, ON, CA), cloned into pGEM-T Easy vectors (Promega, Madison, USA) and sequenced on both strands (University of Valencia, Valencia, Spain). *saGαi1* and *saGαi2* CDSs derived from NCBI automated predictions were also validated by conventional reverse transcription RT-PCR approaches and sequenced as described before. Primer sequences are available in electronic Supplementary Materials Table S2.

### 4.2. Phylogenetic Analyses

Multiple sequence alignments were generated using ClustalX V1.81 (Dublin, Ireland) [89] and Maximum Likelihood (ML) or Neighbor Joining (NJ) phylogenetic trees were constructed for each dataset based on the JTT matrix-based model, using MEGA5 (State College, PA, USA) [90,91] and NJplot (Villeurbanne, France) software’s [92]. Phylogenetic radial view of *Gα* proteins was performed by TreeView (Salisbury, UK) software [93] and cladograms robustness at each branching node was estimated by 1000 random bootstrap replications [94]. The human class (*C*) *G* protein-coupled metabotropic glutamate receptor 1 (*MGR1*, acc.no. AAB05338) was used as a distant out-group for rooting *T1R* phylogenetic trees. 

### 4.3. Generation of Stable pGL3-NFAT-Luc HEK293 Cell Lines

HEK293 cells were cultured in standard medium DMEM (Gibco, Thermo Fisher, Saint Louis, MO, USA) containing 10% FBS, penicillin (100 U/mL) and streptomycin (100 g/mL) at 37 °C, with a humidified atmosphere at 5% CO_2_. pGL3-*NFAT*-luc-HEK293 stable clones were generated by co-transfections of pGL3-*NFAT*-luc and tgCMV/HyTK plasmids (50:1), the latter harboring the hygromycin phosphotransferase gene, using Lipofectamine LTX according to supplier’s protocols (Thermo Fisher, Saint Louis, MO, USA). Initially, HEK293 cells were selected using DMEM containing 400 μg/mL hygromycin B (Sigma, Darmstadt, Germany) in 24-well plates for two weeks; 24 colonies were further grown out in 96-well plates under reduced hygromycin selection (200 µg/mL) for three weeks. Potentially resistant *NFAT*-luc-HEK293 clones (*n* = 22) were validated after incubation in assay medium (DMEM + 1% FBS) containing PMA or Ionomycin (Sigma, Darmstadt, Germany) for 18 h (triplicates, 50,000 cells/well). Luciferase activity was quantified with ONE-Glo™ EX Reagent Kit (Promega, Madison, WI, USA) using a 96-microplate TECAN reader (Trading AG, Switzerland).

### 4.4. Transient Transfections and Stimulation Assays

*saT1Rs* (*saT1R1*, *saT1R2a*, *saT1R2b saT1R3*) and *saGαi1-2* genes were RT-PCR amplified using 5’ and 3’ flanking primers carrying HindIII and XhoI restriction sites, respectively. Gel-purified fragments were cloned into pGEM-T Easy vectors, re-sequenced, digested and subcloned into pcDNA™3 (Invitrogen, Carlsbad, CA, USA). Effective *saT1R* cloning into pcDNA™3 was further verified by HindIII/XhoI digestion. Transient co-transfections were done in 6-well plates (confluence ~70–80%) using 100 μL of reduced serum medium (Opti-MEM, Gibco, Thermo Fisher, Saint Louis, MO, USA*)* containing 150 ng of each selected pcDNA™3 construct*,* 1:10 charge ratio of Enhanced Green Fluorescent expressing construct (EGFP; positive control), and pBluescript II SK (+) vector DNA (Addgene, Watertown, MA, USA) up to 1 µg of totally transfected DNA. Following 24 h incubation, cells were washed with fresh standard medium and plated in polylysine treated plates for additional 24 h. Cells were then stimulated with L-AAs and sugar compounds for 18 h in a reduced assay medium (BME w/o L-glutamate; Gibco, Thermo Fisher, Saint Louis, MO, USA) + 1% FBS), to prevent unspecific Ca^2+^/*NFAT* signaling activation and potential *saT1R* desensitization [95]. All L-AAs and sugars used for in vitro experiments were obtained from Merck KGaA (Darmstadt, Germany).

## 5. Conclusions

This study offers new important information for deciphering the molecular mechanisms underpinning the physiology of fish taste sensory modalities by reporting the first *T1R* gene repertoire in a carnivorous fish species. Additionally, it demonstrates that in vitro cell culture approaches using gene reporter systems based on Ca^2+^ dependent calcineurin/*NFAT* signaling can be readily used to test potency rankings and/or magnitude of *saT1Rs* responses to L-AA tastants and sugars. Through co-transfections of a subset of *saT1Rs* heterodimers, alone or in combination with the G*i* alpha protein subunits *saGαi1* and *sa*G*αi2*, we found that L-AAs induce important taste stimulatory effects differentially mediated by *saT1R1*, *saT1R2a* and *saT1R2b* subunits. Furthermore, we show preliminary evidences that *saGαi* subunits can be involved in both stimulatory and inhibitory *saT1R* transduction signaling mechanisms. Our data strengthen information previously available in herbivorous and omnivorous fish showing that fish species possess multiple putative sweet dimeric receptors (*TR2*n/*TR3*), which are also activated by a broad spectrum of L-AAs with an even higher sensitivity than sugars. It is suggested that the expansion of *TR2* in fish during the third-round genome duplication has provided novel genetic material to facilitate the adaptation to diverse environments and the development of feeding specializations. Finally, our study provides a platform to test *saT1R*-dependent diet selection in one of the most important marine species for Mediterranean aquaculture, and opens new opportunities to further optimize feeds by the use of targeted ingredients or additives susceptible to affect gustatory preferences.

## Figures and Tables

**Table 1:** Evaluation of *saT1R1*/*R3*, *saT1R2a*/*R3* and *saT1R2b*/*R3* sensitivity, as deduced from ligand potency parameter EC50, recorded for 11 L-AAs producing a dose-response curve of activation.

T1R1/R3	EC50	T1R2a/R3	EC50	T1R2b/R3	EC50
AAs	(sensitivity)↓	AAs	(sensitivity)↓	AAs	(sensitivity)↓
**Leu**	1,61 × 10^−4^	**Val**	1,24 × 10^−3^	**Glu**	1,68 × 10^−6^
**Ser**	9,47 × 10^−4^	**Gly**	1,67 × 10^−3^	**Ser**	9,94 × 10^−5^
**Ala**	1,02 × 10^−3^	**Leu**	6,03 × 10^−3^	**Leu**	1,88 × 10^−3^
**Pro**	1,16 × 10^−3^	**Pro**	7,29 × 10^−3^	**Pro**	2,10 × 10^−3^
**Gly**	1,97 × 10^−3^	**Gln**	9,62 × 10^−3^	**Gly**	4,88 × 10^−3^
**Met**	3,77 × 10^−3^	**Thr**	1,96 × 10^−2^	**Ile**	9,55 × 10^−2^
**Ile**	8,06 × 10^−2^	**Met**	4,76 × 10^−2^	**Met**	1,49 × 10^−1^
		**Ile**	1,01 × 10^−1^		

## Supplementary Materials

Supplementary materials can be found at https://www.mdpi.com/1422-0067/21/20/7732/s1.
