# Supplementary material for: Insights into the Function and Evolution of Taste 1 Receptor Gene Family in the Carnivore Fish Gilthead Seabream (Sparus aurata)
Source: Int J Mol Sci. 2020 Oct 19;21(20):7732. doi: 10.3390/ijms21207732 (PMC7594079; doi:10.3390/ijms21207732)
Supplement: Supplementary file 1 [file ijms-21-07732-s001.zip › IJMS_SUPPL.FIG&TABLES&LeEGENDS.docx]

**FIGURE S1: *saT1Rs* MULTIPLE SEQUENCE ALIGNMENTS AND PROTEIN SECONDARY STRUCTURE PREDICTION**

**
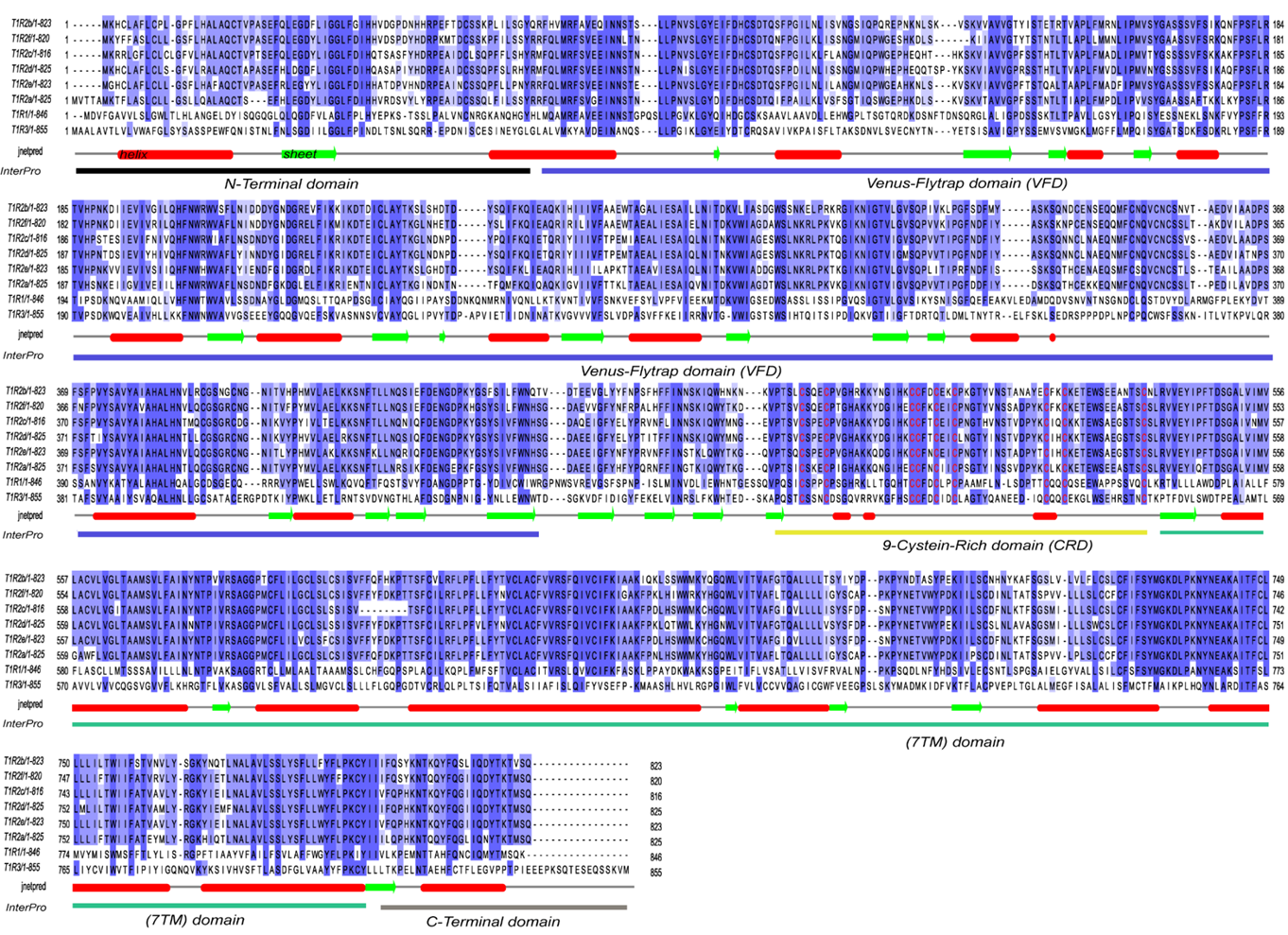
**

Figure S1. Multiple sequence alignments and protein secondary structure prediction of the putative amino acid sequence of *saT1Rs* as deduced by Clustal_X server [**19**]; helices are marked as red tubes, and sheets as light green arrows. Based on *saT1R1* blast for matches against InterPro database signatures (IDs: IPR001828; IPR011500; IPR017978), *saT1Rs* proteins possess the typical structural domains of family 3*(*or C-type*)* GPCRs, including the N-Terminal domain (black line), the Venus Flytrap domain (VFD) (blue line) the 9-Cystein-Rich domain (CRD) (yellow line), the heptahelical transmembrane (7TM) domain (dark green line) and an intracellular carboxyl(C)-Terminal domain (gray line).

**FIGURE S2: GENOMIC STRUCTURE AND TISSUE EXPRESSION ANALYSES OF *saT1R2c* GENE**

**
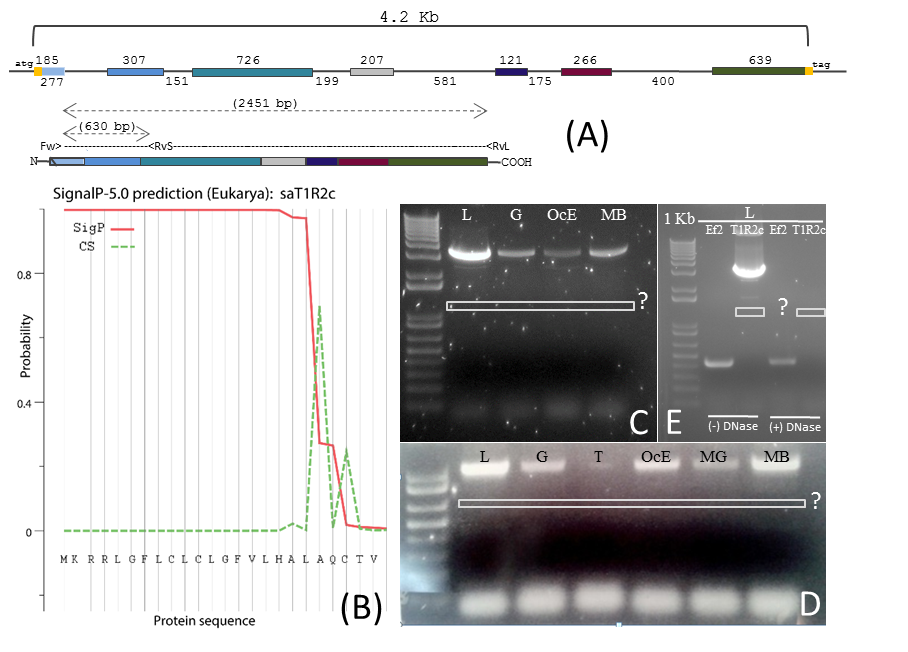
**

Figure S2. *saT1R2*c genomic sequence structurally consists of seven exons ranging from 121 to 726 bp and spanning ⩾4234 bp of the genomic sequence with a predicted CDS of 2451bp (**A**). The deduced *saT1R2c* encoded protein possesses a signal peptide (SigP) and a cleavage site (CS) between position 20 and 21: ALA-QC (Likelihoods of 0.9964and 0.6985, respectively) (**B**). RT-PCR analyses in putative *saT1Rs* highly expressing tissues (minus (-) DNase treated lips (L), gills (G), oral cavity epithelium (OcE) and midbrain (MB) fail to amplify the expected 2451bp transcript (primer pairs: Fw/RvL), producing instead the corresponding genomic fragment size (⩾4234 bp) (**A**, **C**). Similar results were observed using primer pairs encompassing introns 1- 2 (Fw/RvS) which amplify a genomic fragment of 1057 bp and not the expected 630 bp transcript (additional tissues: T, tongue; MG, midgut;**A**, **D**). Depletion of genomic contamination in (+) DNase treated samples confirm absence of *saT1R2c* amplification at both genomic and transcriptional levels (positive controls using Ef2.2,**E;** acc. n: KY388506).

**FIGURE S3: EVALUATION OF L-AA-MEDIATED RISES OF Ca2+ IN EMPTY PCDNA^TM3^ CONSTRUCTS**

**
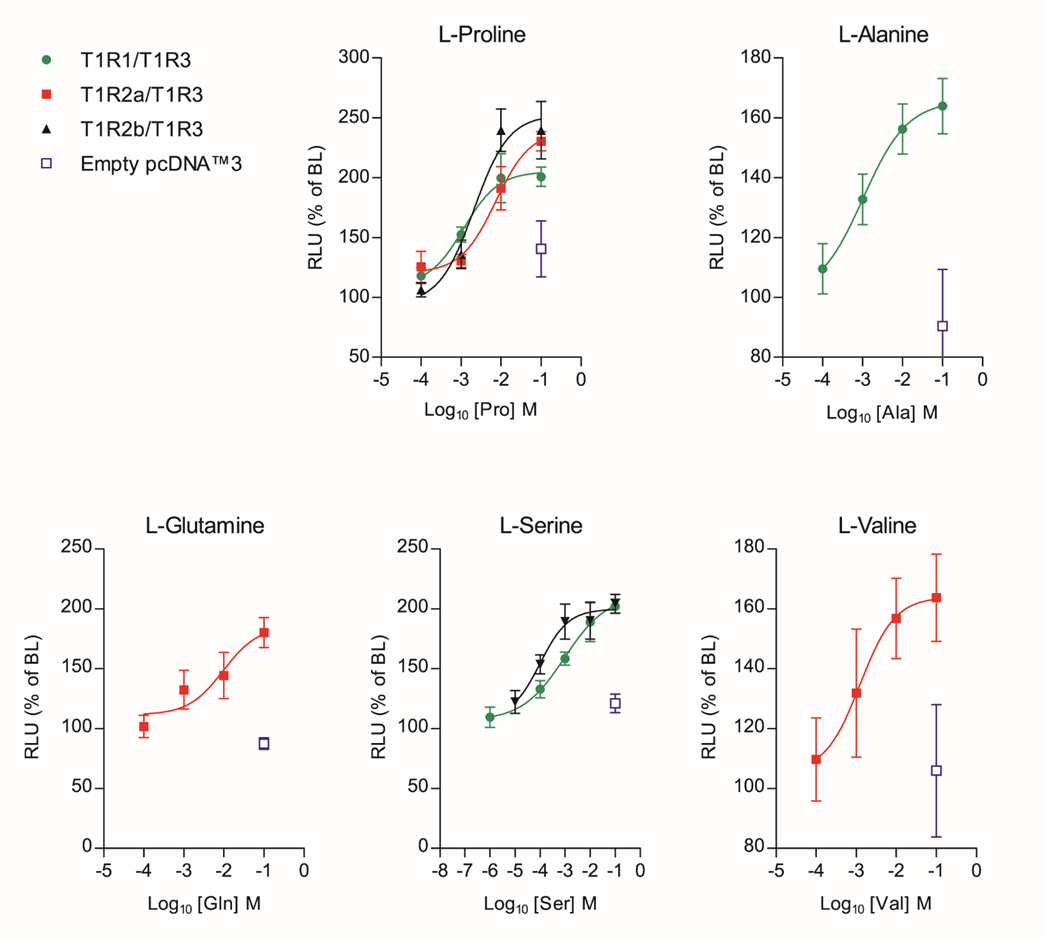
**

Figure S3. Evaluation of L-AA-mediated taste receptor-independent rises of Ca2+ in (Cl.3) cells transfected either with *T1R* heterodimer complexes (dose-responses curves) or with empty pcDNA™3 constructs (blue square). RLU values for emptypcDNA™3 transfections were mean ± SEM of four determinations of Pro, Ala, Gln, Ser, Val MSDs [100mM], normalized to the mean response of same the transfections stimulated with assay medium (BME + 1% FBS), expressed as percentage relative to basal levels (BL).

**TABLE S1: MAXIMUM STIMULATION DOSES (MSD) FOR DIFFERENT L-AAs ON *IN VITRO* EXPRESSED *saT1R* HETERODIMERS**

| AAs | T1R1/R3 [MSD] | T1R1/R3  (RLU) | T1R2a/R3 [MSD] | T1R2a/R3 (RLU) | T1R2b/R3  [MSD] | T1R2b/R3 (RLU) |
| --- | --- | --- | --- | --- | --- | --- |
| Arg | 1,00 × 10^-5^ | 74.44 ± 15.71 | 1,00 × 10^-5^ | 127.95 ± 17.03 | 1,00 × 10^-5^ | 117.68 ± 20.99 |
| Asp | 1,00 × 10^-5^ | 112.96 ± 12.11 | 1,00 × 10^-5^ | 97.23 ± 13.45 | 1,00 × 10^-5^ | 159.75 ± 22.60 |
| His | 1,00 × 10^-5^ | 101.64 ± 14.56 | 1,00 × 10^-5^ | 102.86 ± 11.65 | 1,00 × 10^-5^ | 114.96 ± 10.22 |
| Cys | 1,00 × 10^-4^ | 110.09 ± 17.26 | 1,00 × 10^-4^ | 103.36 ± 13.77 | 1,00 × 10^-4^ | 114.17 ± 11.96 |
| Tyr | 1,00 × 10^-3^ | 105.36 ± 10.44 | 1,00 × 10^-3^ | 123.18 ± 15.75 | 1,00 × 10^-3^ | 123.76 ± 16.06 |
| Trp | 1,00 × 10^-3^ | 111.82 ± 39.66 | 1,00 × 10^-3^ | 219.31 ± 27.45 | 1,00 × 10^-3^ | 140.15 ± 23.63 |
| Lys | 1,00 × 10^-3^ | 108.99 ± 6.78 | 1,00 × 10^-3^ | 131.27 ± 20.79 | 1,00 × 10^-3^ | 146.5 ± 26.79 |
| Glu | 1,00 × 10^-2^ | 212.08 ± 36.35 | 1,00 × 10^-2^ | 154.67 ± 31.51 | 1,00 × 10^-2^ | 181.67 ± 12.40 |
| Phe | 1,00 × 10^-2^ | 192.72 ± 7.84 | 1,00 × 10^-2^ | 201.78 ± 11.95 | 1,00 × 10^-2^ | 207.08 ± 15.68 |
| Asn | 1,00 × 10^-1^ | 128.42 ± 8.58 | 1,00 × 10^-1^ | 136.42 ± 5.72 | 1,00 × 10^-1^ | 157.97 ± 34.03 |
| Val | 1,00 × 10^-1^ | 114.25 ± 26.12 | 1,00 × 10^-1^ | 163.71 ± 20.57 | 1,00 × 10^-1^ | 110.96 ± 14.10 |
| Ile | 1,00 × 10^-1^ | 136.86 ± 22.31 | 1,00 × 10^-1^ | 201.46 ± 16.52 | 1,00 × 10^-1^ | 234.29 ± 34.18 |
| Leu | 1,00 × 10^-1^ | 166.95 ± 16.28 | 1,00 × 10^-1^ | 260.36 ± 30.83 | 1,00 × 10^-1^ | 287.05 ± 21.98 |
| Thr | 1,00 × 10^-1^ | 174.96 ± 31.36 | 1,00 × 10^-1^ | 250.37 ± 24.91 | 1,00 × 10^-1^ | 168.16 ± 42.93 |
| Met | 1,00 × 10^-1^ | 151.96 ± 8.26 | 1,00 × 10^-1^ | 158.93 ± 15.03 | 1,00 × 10^-1^ | 179.08 ± 23.05 |
| Gly | 1,00 × 10^-1^ | 241.17 ± 12.05 | 1,00 × 10^-1^ | 167.68 ± 7.36 | 1,00 × 10^-1^ | 293.94 ± 16.59 |
| Ser | 1,00 × 10^-1^ | 202.11 ± 8.47 | 1,00 × 10^-1^ | 125.34 ± 6.39 | 1,00 × 10^-1^ | 204.30 ± 11.12 |
| Gln | 1,00 × 10^-1^ | 122.42 ± 6.09 | 1,00 × 10^-1^ | 180.11 ± 17.72 | 1,00 × 10^-1^ | 133.04 ± 15.24 |
| Ala | 1,00 × 10^-1^ | 163.90 ± 13.06 | 1,00 × 10^-1^ | 163.83 ± 19.44 | 1,00 × 10^-1^ | 174.83 ± 24.37 |
| Pro | 1,00 × 10^-1^ | 200.76 ±11.44 | 1,00 × 10^-1^ | 230.43 ± 11.32 | 1,00 × 10^-1^ | 239.63 ± 24.95 |

Table S1. Numerical overview of RLU (relative luminescence unit) values based on the mean± SEM of four independent determinations recorded at maximum stimulation doses (MSD) as molarity for the twenty L-AAs tested for each of the three *saT1R* heterodimers.

**TABLE S2: PRIMER SEQUENCES USED FOR CLONING EXPERIMENTS**

**
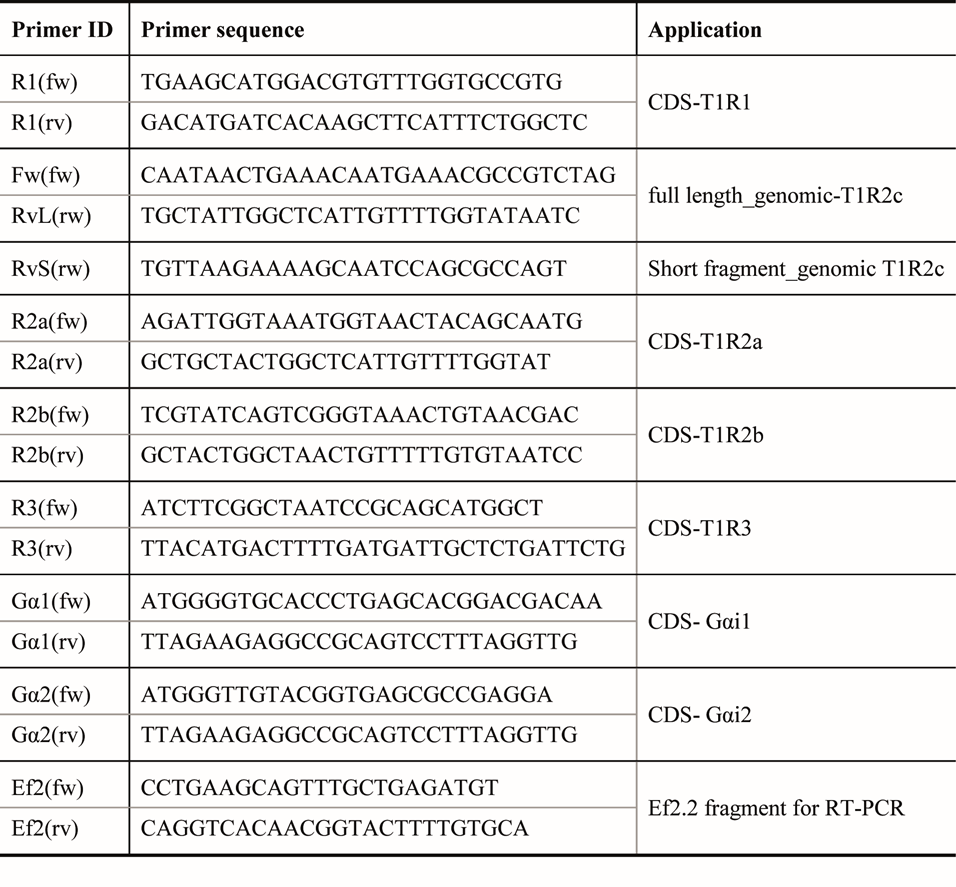
**

Table S2. List of seabream primer sequences in (5'→3') orientation used in the present study
