# Supplementary material for: Insights into the Function and Evolution of Taste 1 Receptor Gene Family in the Carnivore Fish Gilthead Seabream (Sparus aurata)
Source: Int J Mol Sci. 2020 Oct 19;21(20):7732. doi: 10.3390/ijms21207732 (PMC7594079; doi:10.3390/ijms21207732)
Supplement: Supplementary file 1 [file ijms-21-07732-s001.zip › TABLE S1.docx]

| AAs | T1R1/R3 [MSD] | T1R1/R3  (RLU) | T1R2a/R3 [MSD] | T1R2a/R3 (RLU) | T1R2b/R3  [MSD] | T1R2b/R3 (RLU) |
| --- | --- | --- | --- | --- | --- | --- |
| Arg | 1,00 × 10^-5^ | 74.44 ± 15.71 | 1,00 × 10^-5^ | 127.95 ± 17.03 | 1,00 × 10^-5^ | 117.68 ± 20.99 |
| Asp | 1,00 × 10^-5^ | 112.96 ± 12.11 | 1,00 × 10^-5^ | 97.23 ± 13.45 | 1,00 × 10^-5^ | 159.75 ± 22.60 |
| His | 1,00 × 10^-5^ | 101.64 ± 14.56 | 1,00 × 10^-5^ | 102.86 ± 11.65 | 1,00 × 10^-5^ | 114.96 ± 10.22 |
| Cys | 1,00 × 10^-4^ | 110.09 ± 17.26 | 1,00 × 10^-4^ | 103.36 ± 13.77 | 1,00 × 10^-4^ | 114.17 ± 11.96 |
| Tyr | 1,00 × 10^-3^ | 105.36 ± 10.44 | 1,00 × 10^-3^ | 123.18 ± 15.75 | 1,00 × 10^-3^ | 123.76 ± 16.06 |
| Trp | 1,00 × 10^-3^ | 111.82 ± 39.66 | 1,00 × 10^-3^ | 219.31 ± 27.45 | 1,00 × 10^-3^ | 140.15 ± 23.63 |
| Lys | 1,00 × 10^-3^ | 108.99 ± 6.78 | 1,00 × 10^-3^ | 131.27 ± 20.79 | 1,00 × 10^-3^ | 146.5 ± 26.79 |
| Glu | 1,00 × 10^-2^ | 212.08 ± 36.35 | 1,00 × 10^-2^ | 154.67 ± 31.51 | 1,00 × 10^-2^ | 181.67 ± 12.40 |
| Phe | 1,00 × 10^-2^ | 192.72 ± 7.84 | 1,00 × 10^-2^ | 201.78 ± 11.95 | 1,00 × 10^-2^ | 207.08 ± 15.68 |
| Asn | 1,00 × 10^-1^ | 128.42 ± 8.58 | 1,00 × 10^-1^ | 136.42 ± 5.72 | 1,00 × 10^-1^ | 157.97 ± 34.03 |
| Val | 1,00 × 10^-1^ | 114.25 ± 26.12 | 1,00 × 10^-1^ | 163.71 ± 20.57 | 1,00 × 10^-1^ | 110.96 ± 14.10 |
| Ile | 1,00 × 10^-1^ | 136.86 ± 22.31 | 1,00 × 10^-1^ | 201.46 ± 16.52 | 1,00 × 10^-1^ | 234.29 ± 34.18 |
| Leu | 1,00 × 10^-1^ | 166.95 ± 16.28 | 1,00 × 10^-1^ | 260.36 ± 30.83 | 1,00 × 10^-1^ | 287.05 ± 21.98 |
| Thr | 1,00 × 10^-1^ | 174.96 ± 31.36 | 1,00 × 10^-1^ | 250.37 ± 24.91 | 1,00 × 10^-1^ | 168.16 ± 42.93 |
| Met | 1,00 × 10^-1^ | 151.96 ± 8.26 | 1,00 × 10^-1^ | 158.93 ± 15.03 | 1,00 × 10^-1^ | 179.08 ± 23.05 |
| Gly | 1,00 × 10^-1^ | 241.17 ± 12.05 | 1,00 × 10^-1^ | 167.68 ± 7.36 | 1,00 × 10^-1^ | 293.94 ± 16.59 |
| Ser | 1,00 × 10^-1^ | 202.11 ± 8.47 | 1,00 × 10^-1^ | 125.34 ± 6.39 | 1,00 × 10^-1^ | 204.30 ± 11.12 |
| Gln | 1,00 × 10^-1^ | 122.42 ± 6.09 | 1,00 × 10^-1^ | 180.11 ± 17.72 | 1,00 × 10^-1^ | 133.04 ± 15.24 |
| Ala | 1,00 × 10^-1^ | 163.90 ± 13.06 | 1,00 × 10^-1^ | 163.83 ± 19.44 | 1,00 × 10^-1^ | 174.83 ± 24.37 |
| Pro | 1,00 × 10^-1^ | 200.76 ±11.44 | 1,00 × 10^-1^ | 230.43 ± 11.32 | 1,00 × 10^-1^ | 239.63 ± 24.95 |
