# Supplementary figures and images for: Insights into the Function and Evolution of Taste 1 Receptor Gene Family in the Carnivore Fish Gilthead Seabream (Sparus aurata)
Source: Int J Mol Sci. 2020 Oct 19;21(20):7732. doi: 10.3390/ijms21207732 (PMC7594079; doi:10.3390/ijms21207732)

## Slide 1
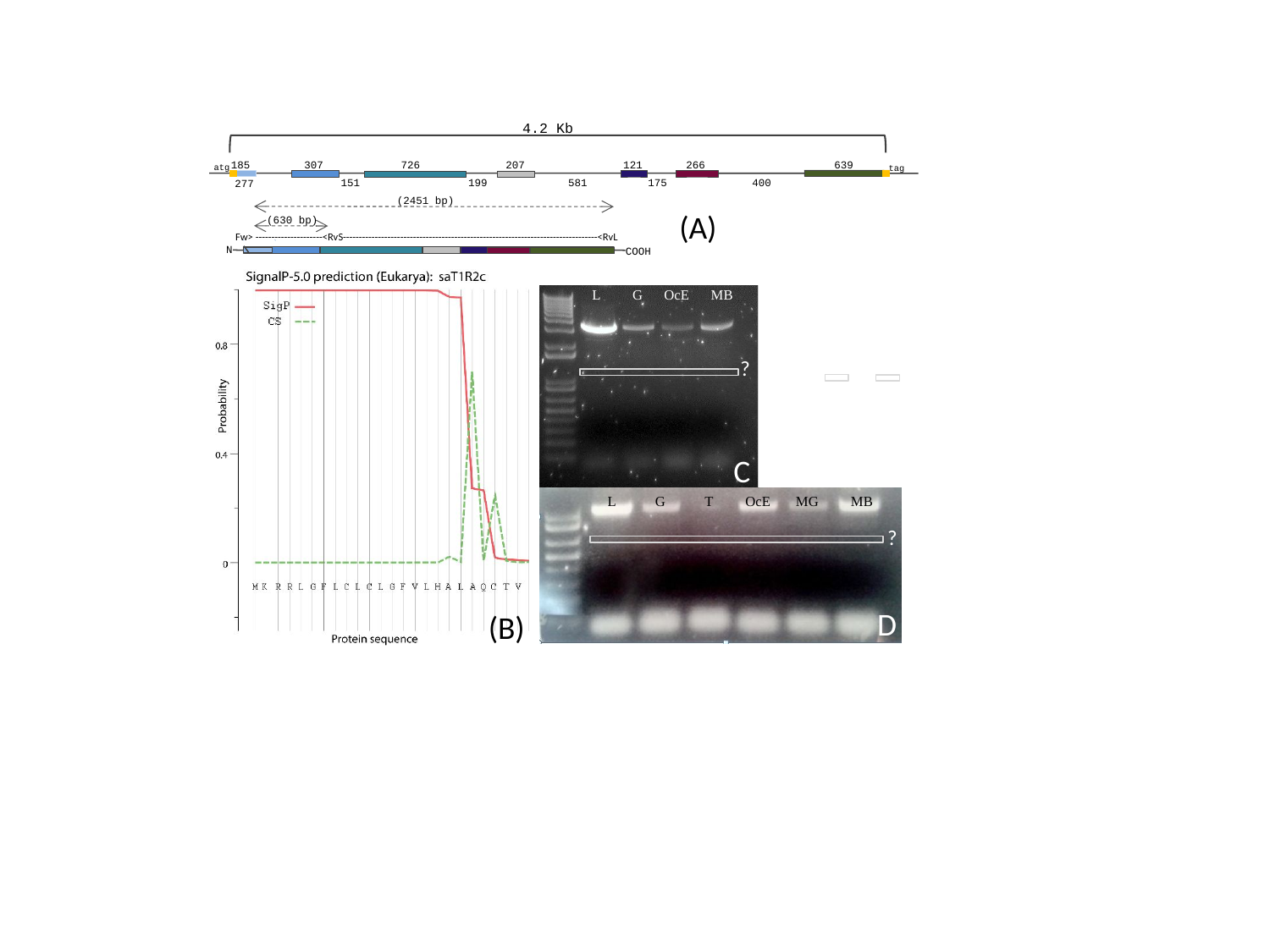

4.2 Kb
185
307
726
207
121
266
639
atg
tag
151
199
581
175
400
277
 (2451 bp)
(A)
 (630 bp)
Fw> ---------------------<RvS--------------------------------------------------------------------------------<RvL
N
COOH
L
L G OcE MB
1 Kb
T1R2c
Ef2
T1R2c
Ef2
?
?
E
C
(-) DNase
(+) DNase
 L G T OcE MG MB
?
D
(B)

Supplement: Supplementary file 1 [file ijms-21-07732-s001.zip › FIG.S2.pptx]
