# Supplementary material for: Insights into the Function and Evolution of Taste 1 Receptor Gene Family in the Carnivore Fish Gilthead Seabream (Sparus aurata)
Source: Int J Mol Sci. 2020 Oct 19;21(20):7732. doi: 10.3390/ijms21207732 (PMC7594079; doi:10.3390/ijms21207732)
Supplement: Supplementary file 1 [file ijms-21-07732-s001.zip › TABLE S2.doc]

| **Primer ID** | **Primer sequence** | **Application** |
| --- | --- | --- |
| R1(fw) | TGAAGCATGGACGTGTTTGGTGCCGTG | CDS-T1R1 |
| R1(rv) | GACATGATCACAAGCTTCATTTCTGGCTC |  |
| Fw(fw) | CAATAACTGAAACAATGAAACGCCGTCTAG | full length_genomic-T1R2c |
| RvL(rw) | TGCTATTGGCTCATTGTTTTGGTATAATC |  |
| RvS(rw) | TGTTAAGAAAAGCAATCCAGCGCCAGT | Short fragment_genomic T1R2c |
| R1(fw) | TGAAGCATGGACGTGTTTGGTGCCGTG | CDS-T1R1 |
| R1(rv) | GACATGATCACAAGCTTCATTTCTGGCTC |  |
| R2a(fw) | AGATTGGTAAATGGTAACTACAGCAATG | CDS-T1R2a |
| R2a(rv) | GCTGCTACTGGCTCATTGTTTTGGTAT |  |
| R2b(fw) | TCGTATCAGTCGGGTAAACTGTAACGAC | CDS-T1R2b |
| R2b(rv) | GCTACTGGCTAACTGTTTTTGTGTAATCC |  |
| R3(fw) | ATCTTCGGCTAATCCGCAGCATGGCT | CDS-T1R3 |
| R3(rv) | TTACATGACTTTTGATGATTGCTCTGATTCTG |  |
| Gα1(fw) | ATGGGGTGCACCCTGAGCACGGACGACAA | CDS- Gαi1 |
| Gα1(rv) | TTAGAAGAGGCCGCAGTCCTTTAGGTTG |  |
| Gα2(fw) | ATGGGTTGTACGGTGAGCGCCGAGGA | CDS- Gαi2 |
| Gα2(rv) | TTAGAAGAGGCCGCAGTCCTTTAGGTTG |  |
| Ef2(fw) | CCTGAAGCAGTTTGCTGAGATGT | Ef2.2 fragment for RT-PCR |
| Ef2(rv) | CAGGTCACAACGGTACTTTTGTGCA |  |

Ef2.2
